# The Influence of Hazelnut Skin Addition on Quality Properties and Antioxidant Activity of Functional Yogurt

**DOI:** 10.3390/foods10112855

**Published:** 2021-11-18

**Authors:** Nayil Dinkçi, Merve Aktaş, Vildan Akdeniz, Alexandrina Sirbu

**Affiliations:** 1 Department of Dairy Technology, Faculty of Agriculture, Ege University, 35100 Izmir, Turkey; nayil.dinkci@ege.edu.tr (N.D.); merveeaktass@gmail.com (M.A.); vildan.akdeniz@ege.edu.tr (V.A.); 2 Faculty of MMAE Ramnicu Valcea, Constantin Brancoveanu University of Pitesti, 240210 Ramnicu Valcea, Romania

**Keywords:** waste valorisation, hazelnut skin, functional yogurt, antioxidant activity

## Abstract

There is an increasing interest in applying fruit-processing wastes as functional food ingredients. Hazelnut skin, an interesting and innovative ingredient has recently been evaluated as one of the richest edible sources of polyphenolic compounds. This study aimed to evaluate the use of hazelnut skin as a functional additive in yogurt and to determine the effect of various percentages (2%, 3%, and 4%) of hazelnut skin on the physicochemical, microbiological, rheological, biochemical, and sensorial properties of yogurt. The results showed that the addition of hazelnut skin significantly increased total solids from 16.5% to 17.7% and fat content from 3.45% to 4.60% and decreased titratable acidity by up to 36%. The enrichment with hazelnut skin also improved the viability of yogurt bacteria, water holding capacity (WHC), and antioxidant activity of yogurts. Better results for WHC and antioxidant values were found in yogurt enriched with 4% hazelnut skin. Total phenolic content and Fe^2+^ chelating activity of yogurts increased with the increasing hazelnut skin ratio. However, yogurts with hazelnut skin had low instrumental texture parameters and apparent viscosity values. On the other hand, acceptable sensorial properties similar to control yogurts increase the consumption potential of yogurts enriched with hazelnut skin.

## 1. Introduction

The increase in the global human population and the inefficient use of natural resources threaten sustainable food production. In addition, by-products resulting from waste generation are among the most important issues of the food industry [1]. Most of these by-products have significant potential in developing new and sustainable functional foods due to their nutritional properties [2]. Thus, the valorisation of by-products is both necessary and important in many respects, including functional new product development, environment protection, and economic development [1].

One of these by-products is hazelnut skin. Hazelnut (*Corylus avellana* L.) is one of the most important nuts worldwide due to its flavour, fat-soluble bioactives, good level of dietary fibre, phenolic compounds/phytochemicals, nutritional value comprising a rich variety monounsaturated fats and vitamins, and high content of minerals; it has wide applications in the confectionery industry as well as in dairy, bakery, and chocolate products [3,4]. In 2003, the U.S. Food and Drug Administration (FDA) recognized nuts as ‘heart-healthy’ food. Hazelnuts are an excellent source of antioxidants including phenolic and hydroxycinnamic acids such as gallic acid, caffeic acid, protocatechuic acid, vanillic acid, p-coumaric acid, ferulic acid, sinapic acid and flavonoids such as catechin, quercetin, myricetin, and kaempferol [5]. The results of the studies showed that hazelnut is one of the best sources of combined polyphenols and total fibre [6,7].

According to the FAO, 1,125,178 tonnes of hazelnuts with shells were produced in the world in 2019. Turkey is the leading global manufacturer of hazelnuts, with 69% of total production, followed by the European Union countries and the USA [8]. Hazelnut skin is discarded upon the roasting process of the hazelnuts. The amount of hazelnut skin is about 2.5% of the total kernel weight and it is the main by-product after roasting [6]. Hazelnut skin, which is the part of the hazelnut with the highest antioxidant activity, is a rich source of phenolic compounds and also dietary fibre [3,9]. It prevents the oxidation of low-density lipoprotein in humans and also helps enhance host gastrointestinal health by promoting a beneficial microbiota profile; thus, it exerts a significant role in the prevention of many diseases, including cardiovascular diseases, cancers, neurodegenerative diseases, diabetes, and osteoporosis [7]. It is a valuable by-product due to these properties, and there are studies reporting that this waste material could find application as a functional food ingredient [4].

Nowadays, consumers consider that their health is directly related to the foods consumed, and therefore functional foods are in demand by health-conscious consumers. Food production methods also change significantly to meet consumer demands. A pathway for developing functional food is adding the ingredients containing bioactive peptides, phytochemicals, omega 3 polyunsaturated fatty acids, dietary fibres, probiotics, and prebiotics to food during production [10].

Yogurt is a popular dairy product whose consumption and production rate is still increasing worldwide. Recently, it became one of the foods whose functional properties are developed by adding functional ingredients, including walnut, hazelnut, almond, pistachio, or fibre-rich by-products such as coat/peel and seed powder of different fruits (pineapple, passion fruit, etc.) [1,11,12,13,14]. It is an essential nutrient with enormous nutritional and health benefits, containing calcium (absorbed better from yogurt than milk), high-quality proteins and peptides, phosphorus, B vitamins, and many other beneficial substances for both children and adults [5]. However, yogurt with improved functional properties such as antioxidant and antimicrobial effects has a positive effect on many diseases, including coronary heart disease, cancer, osteoporosis, and food allergies [5,13].

In this context, the combination of yogurt and hazelnut skin could possibly develop a functional food with a wide range of beneficial effects. The latest research results indicate that hazelnut skin deserves more interest as a valuable raw material of functional food due to the content of phenolic compounds with antioxidant properties and dietary fibre [4,6,9]. Del Rio et al. (2011) [9] reported that the total antioxidant capacity of hazelnut skin was about three times that of whole walnuts, seven to eight times that of dark chocolate, 10 times that of espresso coffee, and 25 times that of blackberries. Antioxidants are markedly important compounds in food science due to their ability to prevent lipid oxidation in foodstuffs and decrease the negative effects of reactive oxygen species on physiological functions in humans. Polyphenols, which are commonly found in plants, have become one of the most studied natural antioxidants, with the increasing preference of consumers for natural products [6]. They can lower the oxidation and inflammation processes that may trigger many pathological conditions or age-related chronic diseases by acting synergistically with other phytochemicals [5]. In addition, the incorporation of hazelnut skin, which is a fibre-rich by-product, into yogurts represents means of augmenting the consumption of fibre, owing to the popularity of this foodstuff. Studies have shown that high-fibre yogurt consumption has health benefits, such as reduction in the risk of obesity, diabetes, cancer, hypertension, hypercholesterolemia, and cardiovascular and gastrointestinal disorders and also promotes intestinal microflora and gastrointestinal immunity [15,16]. As fibre deficiency in the diet may cause many nutrition-related diseases, an average daily intake of 25 g of fibre is recommended by the European Food Safety Authority [17]. The addition of various dietary fibres into yogurts as a functional component also positively affects the rheological properties of yogurts and contributes to the reduction of the energy value of the final product. Besides consumers’ preference for healthy foods with high dietary fibre content and low calorie value, food manufacturers are also concerned with new sources of dietary fibre obtained from natural sources as ingredients [18,19].

This study was carried out to develop a functional product by the superior valorisation of a food industry waste, namely hazelnut skin in yogurt as a source of dietary fibre and antioxidants. Thus, while reducing industrial wastes, it will also contribute to human health and the circular food system. The addition of hazelnut skin before the fermentation process, which is also important for the yogurt producers, distinguishes our study from similar studies [13]. To the best of our knowledge, the information on this topic is still limited, and there are only a few relevant studies.

## 2. Materials and Methods

### 2.1. Bacterial Cultures, Raw Cow’s Milk, and Hazelnut Skin

The commercial yogurt starter culture containing *Streptococcus thermophilus* and *Lactobacillus delbrueckii* subsp. *bulgaricus* (Wisby-Danisco yogurt culture-Yo-Mıx 500) in freeze-dried direct vat set (DVS) form was used in the study. The cow’s milk was provided by Ege University, i.e., the Pilot Dairy Plant of the Agricultural Faculty’s Department of Dairy Technology. Non-fat milk powder was supplied by Pınar Sut A.S (İzmir, Turkey). Hazelnut skin was obtained from Gürsoy Tarımsal Ürünler Gıda Sanayi ve Ticaret AŞ (Ordu, Turkey). The provided hazelnut skins were kept in vacuum packages at −20 °C until the production and ground with a domestic electric coffee grinder just before use.

### 2.2. Manufacture of Yogurt

Yogurt samples were manufactured from whole milk containing 11.93 ± 0.02% TS, 3.10 ± 0.00% protein, 0.16 ± 0.00% lactic acid, 6.75 ± 0.00 pH, and 3.50 ± 0.00% fat. The non-fat milk solids were increased to 13% by skim milk powder at 40 °C. The milk was divided into four equal parts: the control group (K) without hazelnut skins and the other groups enriched with 2% (A), 3% (B), and 4% (C) hazelnut skins. After they were mixed properly, each milk group was heated at 85 °C for 30 min and cooled to 45 °C for incubation. The yogurt culture was added to the milk. All cultures were used according to the manufacturer’s instructions. The mixtures were put into 200 g and 80 g plastic containers and incubated at 42 °C until pH 4.65 was reached. Following incubation, yogurt samples were cooled down and stored at 4 °C over 28 days for the physicochemical, microbiological, rheological, biochemical, and sensorial analyses. The analyses were carried out on day 1, 7, 14, 21 and 28 of storage. The experiments were replicated two times.

### 2.3. Physicochemical Analysis

The total solid (TS) and protein content were determined according to the methods described in the Association of Official Analytical Chemists methods [20]. Fat contents were analysed by the Gerber method [21]. The pH of the yogurts was determined using a pH meter (Hanna Instruments, Woonsocket, RI, USA). Titratable acidity was expressed as g of lactic acid/100 g. Since the colour change in yogurts with hazelnut skin cannot be distinguished visually, the titration with 0.1 N sodium hydroxide after adding 1 mL of 1% phenolphthalein was carried out in the presence of a pH meter until reaching the end point, pH = 8.1 [20].

### 2.4. Microbiological Analysis

Samples (10 mL) were diluted to 100 mL with ringer solutions. Afterwards, serial dilutions were prepared, and bacterial enumerations were performed using pour plate technique. *Streptococcus thermophilus* was enumerated using M17 agar at 37 °C for 72 h aerobically [22], and *Lactobacillus delbrueckii* subsp. *bulgaricus* was enumerated using MRS agar (pH 5.2 and anaerobic incubation) at 42 °C for 48 h [23].

### 2.5. Rheological and Other Physical Analysis

#### 2.5.1. Hardness, Adhesive Force, Consistency, and Viscosity Index

The hardness, adhesive force, consistency, and viscosity index of yogurts were determined with the Brookfield CT 3 Texture Analyzer (Middleboro, MA, USA) device and TA4/1000 acrylic cylinder probe (probe diameter: 38.1 mm) using a single-compression cycle test (Device settings: target value: 4500 g, trigger load: 4.5 g, test speed: 1 mm/s, depth of the sample: 57 mm, probe penetration: 15 mm). The values were obtained using a Brookfield Texture Pro CT software program V 1.2. The test was carried out at 4 °C after removing the samples from the refrigerator.

#### 2.5.2. Apparent Viscosity

The apparent viscosity of the yogurts was measured after stirring the product for 20 s, using the Brookfield DV-II Pro Model viscometer (Middleboro, MA, USA) with the appropriate spindle and spindle rotation selection (Spindle no: 3, 20 rpm) at 4 °C according to Brookfield Texture Application Note—Yogurt. The results were measured in mPas, after 35 s of shearing, paying attention to the torque values being between 10 and 100%. The apparent viscosity values of yogurts were recorded using RHEOCALC 32 Application Software (Brookfield Engineering Laboratories Inc., Middleboro, MA, USA).

#### 2.5.3. Water Holding Capacity

The water holding capacity (WHC) of yogurts was determined according to a method described by Isanga and Guonong (2009) [24], with minor modifications. A sample of about 15 g of yogurt was transferred to a centrifuge tube and centrifuged for 15 min at 8000× *g* and 4 °C. The resultant supernatant was removed, and the separated whey was weighed. The water holding capacity was calculated as a percentage (%) from the following formula:

$$\mathrm{WHC}\;\left(\%\right)=1-\frac{\mathrm{Separated}\;\mathrm{Whey}\;\mathrm{Weight}\;\left(g\right)\times 100}{\mathrm{Yogurt}\;\mathrm{Weight}\;\left(g\right)}$$

#### 2.5.4. Colour Analysis

The colour values of yogurt samples were determined as a Hunter scale using a Minolta CR−400 colorimeter (Minolta, Osaka, Japan). The L*, a* and b* colour dimensions were read for the yogurt samples. The Hunter L* value represents lightness from black (0) to white (100), a* value represents colour ranging from red (+) to green (−), and the b* value represents yellow (+) to blue (−). Chroma (c*) value, which represents the intensity of the colour, was calculated from the formula below [25]:

$$c*=\sqrt{{a*}^{2}+{b*}^{2}}$$

Delta E (ΔE) was calculated to measure changes in the visual perception of the colour of the sample compared to the control using the following formula [26,27]:

$$\Delta E=\sqrt{{\left(L_{1}-L_{2}\right)}^{2}+{\left(a_{1}-a_{2}\right)}^{2}+{\left(b_{1}-b_{2}\right)}^{2}}$$

ΔE = Difference in colour measurement between the two samples.

#### 2.5.5. Microstructure

The microstructure of yogurt samples was determined with a digital camera (Nikon, Tokyo, Japan, Coolpix 4500) connected to a light microscope (Olympus, Tokyo, Japan, 4×). Then, the images were edited using the Adobe Photoshop Elements program.

### 2.6. Biochemical Analysis

#### 2.6.1. Proteolytic Activity

The ο-phthaldialdehyde (OPA) method described by Donkor et al. (2006) [28] was used to determine the proteolytic activity.

#### 2.6.2. Antioxidant Activity

The antioxidant activities of yogurt samples were determined by measuring total phenolic content and Fe^2+^ chelating activity.

The total phenolic content (TPC) of yogurt samples was determined with a modified method adapted from Apostolidis et al. (2007) [29]. A total of 20 g of yogurt samples was taken into centrifuge tubes, and 20 mL of distilled water was added and homogenized for 2 min. Homogenized samples were centrifuged at 10,000 rpm for 10 min, and then 0.5 mL of the extract was taken. Then, 1 mL of 95% ethyl alcohol and 5 mL of distilled water were added to the extract and mixed. In addition, 0.5 mL of 50% folin was added and mixed again. After leaving 5 min at room temperature, 1 mL of 5% sodium carbonate (Na_2_CO_3_) was added and incubated for 60 min at room temperature. Then, the absorbance of the solution was measured at 725 nm. Gallic acid was used at different concentrations as a reference.

The chelating activity of yogurt samples on Fe^2+^ was measured according to a method modified from Unal et al. (2013) [30]. One millilitre of sample (1 g/mL) was mixed with 3.7 mL deionized water and incubated with 0.1 mL FeCl_2_4H_2_O (2.0 mM) for 5, 10, 30, and 60 min. After incubation, 0.2 mL ferrozine (5.0 mM) was added to the solutions and left at room temperature for 10 min. Then, the absorbance of the solution was measured at 562 nm. The control was prepared using water instead of sample following the same method. EDTA solution (0.1 mg/mL) was also used in the same way for comparison. The chelating activity of the samples was calculated by the following formula:

$${\mathrm{Fe}}^{2+}\mathrm{chelating}\;\mathrm{activity}\;(\%)=[1-(\mathrm{absorbance}\;\mathrm{of}\;\mathrm{sample}/\mathrm{absorbance}\;\mathrm{of}\;\mathrm{control})]\times 100$$

### 2.7. Sensorial Analysis

The yogurt samples were evaluated according to the method modified from Akalın et al. (2012) [31]. Sensory evaluation of the samples was performed by a panel group of ten academics from the Department of Dairy Technology (Ege University, Izmir, Turkey), who were well experienced and familiar with yogurt. The yogurt samples were graded for five sensory attributes, including appearance, aroma, taste, texture, and overall acceptability, and the samples were scored based on 5-point hedonic scales (1: dislike extremely; 5: like extremely).

### 2.8. Statistical Analysis

In the research, two experimental replicates were carried out. The data obtained were processed by an ANOVA, and the mean differences were analysed using Duncan’s multiple range test at the pre-set *p* < 0.05 level using SAS software (version 8; SAS Institute Inc., Cary, NC, USA).

## 3. Results

### 3.1. Physicochemical Properties

Table 1 shows some physicochemical components of yogurts, such as fat, TS, and protein. As shown in Table 1, the average composition of yogurts revealed that enrichment with hazelnut skin caused a significant difference (*p* < 0.05) in the fat content of samples. In addition, the TS of the yogurts varied from 16.49 ± 0.07% to 17.68 ± 0.12%. However, enrichment with hazelnut skin did not cause a significant difference (*p* > 0.05) in the protein content of yogurts.

Table 2 shows the changes in pH and titratable acidity expressed as lactic acid of yogurts during 28 d of storage at 4 °C. Both enrichment with hazelnut skin and storage time significantly affected the pH values of yogurts (*p* < 0.05).

### 3.2. Microbiological Properties: Lactic Acid Bacteria Viability

The microbiological attributes of yogurt samples during the storage period are presented in Table 2. Bacteria viability was significantly influenced by both the storage time and hazelnut skin enrichment (*p* < 0.05).

*Lactobacillus delbrueckii* subsp. *bulgaricus* count at the beginning of storage varied between 8.61 and 9.18 log CFU/g, while it varied between 8.52 and 8.77 log CFU/g at the end of storage. During the whole storage period, *Streptococcus thermophilus* (*S. thermophilus*) count ranged from 8.30 to 8.97 log CFU/g (Table 2). *S. thermophilus* counts reached their highest value on day 21 of storage for all yogurt groups, except yogurts enriched with 3% hazelnut skin (B). For this group the highest value was obtained on day 14.

### 3.3. Rheological Properties

The instrumental texture parameters (hardness, adhesive force, consistency, and viscosity index) of the yogurts are shown in Table 3.

The difference in viscosity index value between control and enriched yogurts was mostly not statistically significant (*p* > 0.05). However, a significant effect was observed for the hardness, adhesive force, and consistency of yogurts enriched with the different amounts of hazelnut skins. The difference between these values was statistically significant for most storage days (*p* < 0.05). These values also decreased as the amount of hazelnut skin in yogurts increased.

Viscosity is an important parameter for the determination of consumer acceptability, yogurt quality, and curd stability. Table 3 shows the apparent viscosity values of the yogurt samples. The highest initial and final apparent viscosity values were determined in the control sample, while the highest values were in yogurts enriched with 1% hazelnut skin on days 14 and 21. However, the differences between the control and group A with 1% hazelnut skin were not found to be statistically significant during storage (*p* > 0.05).

### 3.4. Water Holding Capacity (WHC)

**Figure 1:**
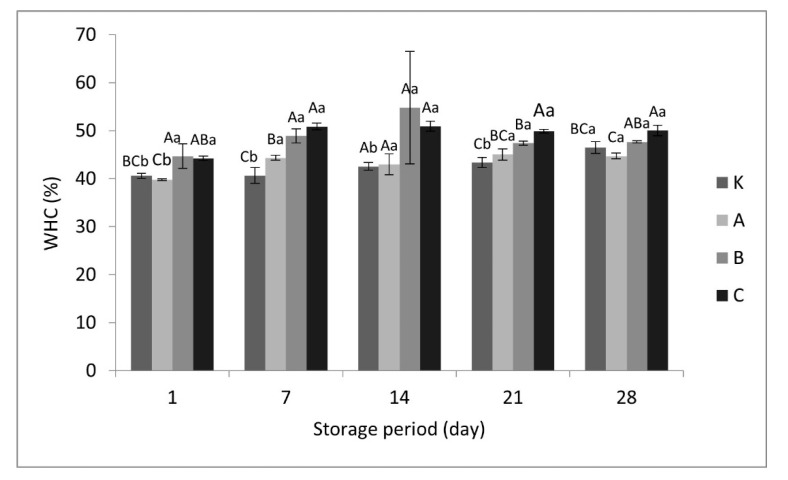
Water holding capacity (WHC) values in yogurts enriched with hazelnut skin during storage (Legend: ^a–b^ Means ± standard deviations in the same yogurt group with different superscript lowercase letters are significantly different (*p* < 0.05); ^A–C^ Means ± standard deviations for the same storage day with different superscript uppercase letters are significantly different (*p* < 0.05)).

Syneresis is one of the most important structural and textural defects in yogurts. Thus, WHC is an important quality criterion, which is related to the ability of proteins for water-keeping within the yogurt curd. The WHC values in yogurt samples are shown in Figure 1, where the error bars indicate standard deviations (the level of significance was pre-set at *p <* 0.05). Control yogurts exhibited the lowest levels of WHC throughout the storage, whereas the highest levels of WHC were obtained by yogurts enriched with more than 2% hazelnut skin (i.e., with 3% hazelnut skin on day and 14 and with 4% hazelnut skin on the other days: 7, 21, and 28). The differences between groups were generally found to be significant during storage (*p* < 0.05).

### 3.5. Colour

Colour is an important quality criterion; it affects consumer preference as it is the first property perceived by the consumers. Colour-space parameters (L*, a*, b* and Chroma (C*)) of yogurts are presented in Table 4. Colour intensity (C*) increased with colour perception, shifting towards red due to hazelnut skin enrichment. It was observed that the yogurts enriched with hazelnut skin were darker. The ΔE value between the control group and the C group enriched with 4% hazelnut skin showed the highest values during storage. The highest value (35.19 ± 0.69) was obtained for the C sample on day 21.

### 3.6. Microstructure

**Figure 2:**
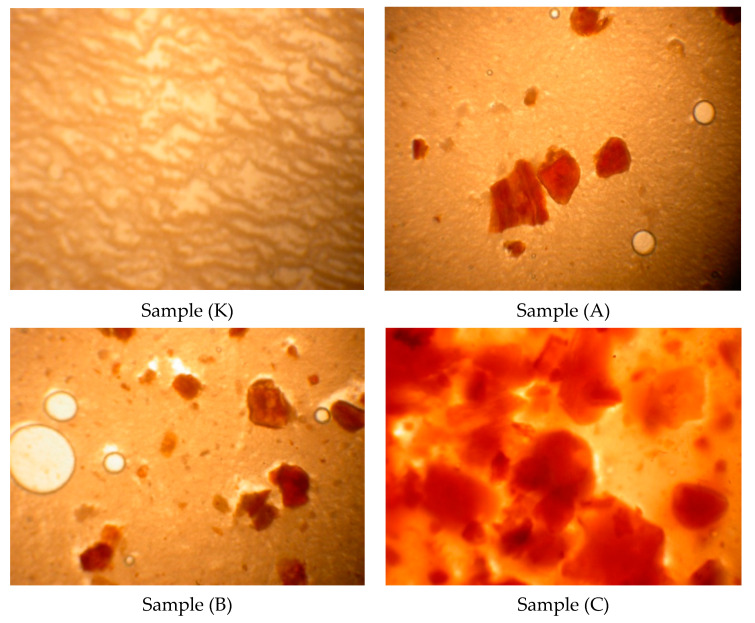
Microstructures of yogurts enriched with hazelnut skin during storage. Sample (K) control yogurt without hazelnut skin; Sample (A) yogurt enriched with 2% hazelnut skin; Sample (B) yogurt enriched with 3% hazelnut skin; Sample (C) yogurt enriched with 4% hazelnut skin.

The microstructures of yogurt samples with different amounts of hazelnut skin are given in Figure 2. The density in the structure of yogurts enriched with hazelnut skin, especially containing 4% hazelnut skin, is visible compared to control yogurts.

### 3.7. Biochemical Properties

#### 3.7.1. Proteolytic Activity

**Figure 3:**
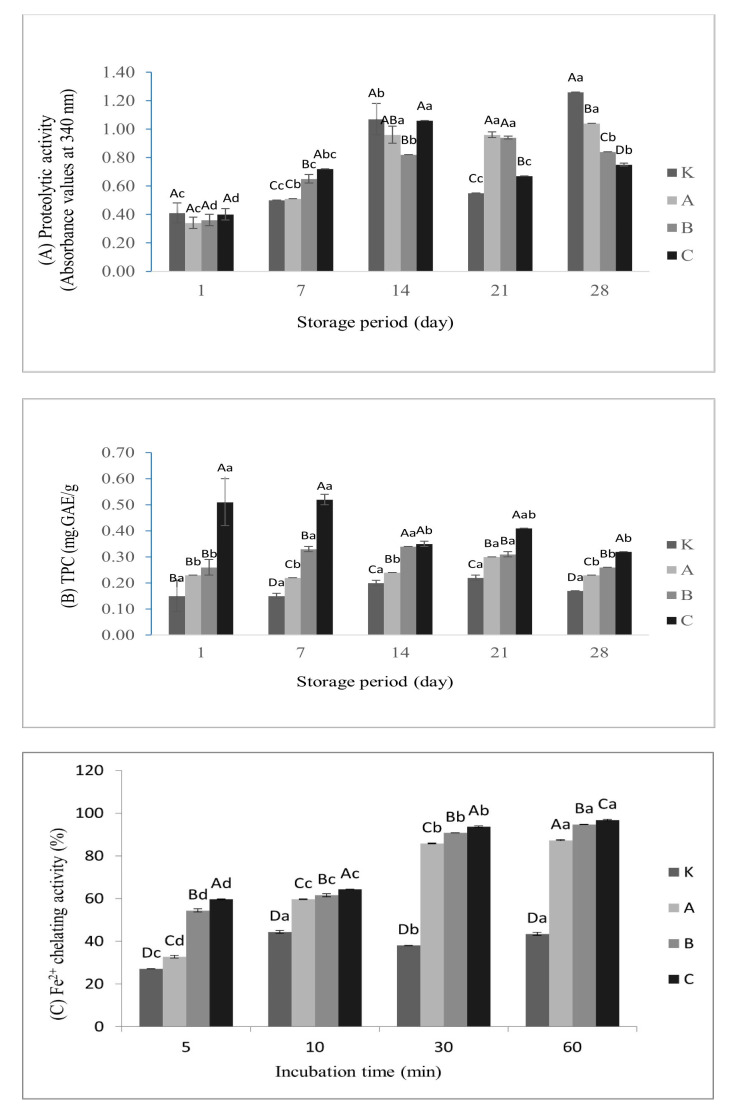
(**A**) Proteolytic activity, (**B**) TPC and (**C**) Fe^2+^ chelating activity in yogurts enriched with hazelnut skin (Legend: ^a–d^ Means ± standard deviations in the same yogurt group with different superscript lowercase letters are significantly different (*p* < 0.05); ^A–D^ Means ± standard deviations for the same storage day with different superscript uppercase letters are significantly different (*p* < 0.05).

Despite the weak proteolytic activity, lactic acid bacteria cause significant proteolysis in yogurt due to the symbiotic effect [32]. Figure 3 represents the proteolytic activities in yogurts. Enrichment with hazelnut skin and storage time affected significantly (*p* < 0.05) the proteolytic activity of yogurts, except for day 1.

#### 3.7.2. Antioxidant Activity

Figure 3 shows the TPC and Fe^2+^ chelating activity of the yogurts. The enrichment of yogurts with hazelnut skin showed significant differences (*p* < 0.05) in TPC compared to control yogurts during the whole storage period. In addition, an increasing trend of Fe2+ chelating activity was registered in accordance with an increase in both amounts of hazelnut skin added in yogurts and incubation time.

### 3.8. Sensorial Properties

**Figure 4:**
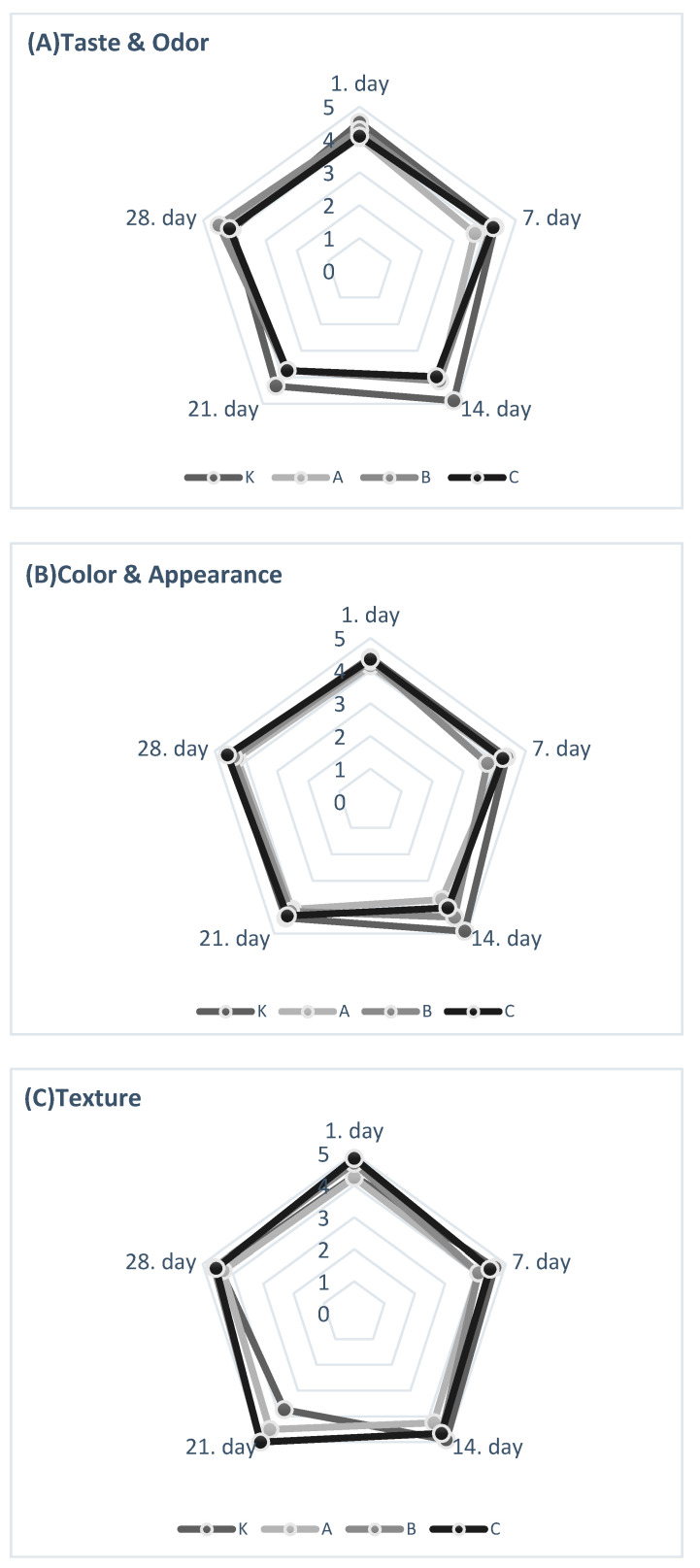
Sensory evaluation of yogurts during storage: (**A**) Taste and Odour, (**B**) Colour and Appearance, and (**C**) Texture. (Legend: The yogurts were scored based on 5-point hedonic scales: 1—dislike extremely; 5—like extremely).

Scores recorded for taste-smell, colour-appearance, and texture are shown in Figure 4. The results indicated that both the enrichment of hazelnut skin and storage time generally had no significant effect on sensory properties, with a few exceptions.

## 4. Discussion

As is the case in all fermented dairy products, the composition of the raw milk used in yogurt production affects the quality of yogurts. As expected, there was an increase (*p* < 0.05) in the fat content of the yogurts enriched with hazelnut skin. As the hazelnut skin ratio increased, the fat content of yogurts increased as a consequence of the oil contained in the hazelnut skin. As the rate of hazelnut skin increased, total solids also increased. However, statistically significant (*p* < 0.05) differences were found only between the control and group C, which contained 4% hazelnut skin.

In general, the pH values of yogurts enriched with hazelnut skin were higher than control yogurts, and these differences were found to be statistically significant (*p* < 0.05) between the control and C groups. The results are consistent with those published by Buniowska et al. (2020) [16], who found the highest pH value in yogurt enriched with 3% spelt hull, and the lowest in the control. In addition, Ozturkoglu-Budak et al. (2016) [11] obtained similar results and indicated that all dried nut- (walnut, hazelnut, almond, or pistachio) fortified yogurts had higher pH values than that of the control yogurt. Otherwise, while the pH values of groups B and C tended to decrease during storage, the decrease in the control group turned into an increase on the last storage day. The decrease in the pH of fermented dairy products during storage has been reported by many researchers [31]; on the other hand, some researchers have observed an increase in pH during storage. This increase may be due to the ability of *S. thermophilus* to produce some basic metabolites during the later stages of storage [31]. Depending on the increase in the pH value of the control yogurt on day 28, no significant difference (*p* > 0.05) was detected among all samples on the last storage day.

Although some fluctuations were observed in the lactic acid values of all samples during storage, in general, the titratable acidity decreased as the amount of hazelnut skin in yogurts increased. This situation is also compatible with the pH values of yogurts. In another study, the yogurts enriched with buckwheat and spelt hull had lower total acidity than that of the control, and the increase in the dose caused even lower acidity [16]. The higher pH and lower acidity obtained in the current study could be due to the dietary fibre coming from hazelnut skin.

Regarding the microbiological aspects, fluctuations in *Lactobacillus delbrueckii* subsp. *bulgaricus* (*L. delbrueckii* subsp. *bulgaricus*) counts during storage were registered. However, values at the end of storage were lower than the values at the beginning of storage, except for yogurt enriched with 2% hazelnut skin. In this group, the lowest value was detected at the beginning of storage. However, this value is the lowest value enumerated during the whole storage period. *L. delbrueckii* subsp. *bulgaricus* counts reached their highest value on day 21 of storage for all yogurt groups. In addition, the highest viable counts of *L. delbrueckii* subsp. *bulgaricus* were enumerated in yogurts enriched with 4% hazelnut skin on day 21. Generally, enrichment with hazelnut skin improved counts of *L. delbrueckii* subsp. *bulgaricus* in yogurts during storage. Our results are in agreement with the observations of other authors, who found yogurts enriched with hazelnut skin had higher *L. delbrueckii* subsp. *bulgaricus* counts than controls [6]. This is likely due to the prebiotic effect of fibres introduced in yogurts through hazelnut skin. Other works reported that *L. delbrueckii* subsp. *bulgaricus* showed a significant increase in viability in the presence of dietary fibre in yogurts [33] and that probiotic yogurts enriched with lemon and orange fibres had higher *L. delbrueckii* subsp. *bulgaricus* counts than control yogurts [32].

During the whole storage period, the highest viable counts were enumerated in yogurts enriched with 4% hazelnut skin on day 21 for *S. thermophilus* as well as for *L. delbrueckii* subsp. *bulgaricus.* Overall, *S. thermophilus* counts in enriched yogurts reached higher values than the control, with the effect being obvious at the highest added percent (4%) of hazelnut skin to yogurt. Similar results were obtained by other authors, who indicated either that yogurts enriched with hazelnut skin had higher *S. thermophilus* counts than the control [6] or that enrichment with dietary fibre increased the viability of *S. thermophilus* in yogurts and probiotic yogurts [32,33].

The highest hardness, adhesive force, and consistency values during the whole storage period were registered for the control yogurts, while yogurts enriched with 4% hazelnut skin had the lowest values. Lower instrumental texture parameters of enriched yogurts were previously observed in other studies, in which different types of by-products were used. The use of pineapple peel powder [12] and the use of spelt and buckwheat hull at a dose of 1.5% [16] also resulted in lower hardness of yogurt. This may be due to a weak gel, attributed to thermodynamic incompatibility between milk proteins and polysaccharides from by-products.

Concerning the apparent viscosity, the lowest viscosity values were in yogurts enriched with 4% hazelnut skin, and the differences between the control and group C (with 4% hazelnut skin) were statistically significant during storage, except for day 21 (*p* < 0.05). In general, this result was consistent with the hardness and consistency values. In agreement with the results found in this study, Demirci et al. (2017) [34] stated that the addition of rice bran to yogurts decreased the viscosity of yogurts. Moreover, others reported that increasing the concentration of pomegranate peel extracts, the viscosity values decreased in yogurts; this result may be due to the effect of pomegranate peel extract on the aggregation of the network in yogurts via electrostatic interactions [35]. Several studies also showed that supplementation with different dietary fibres increased viscosity in yogurts [13,24]. The soluble and insoluble nature of dietary fibres change their effect on viscosity. Water soluble fibres are the main component that increases the viscosity. The reason for the differences in the studies may be due to the proportion, amount, and size of the soluble or insoluble part in the fibre used [19].

Enrichment with hazelnut skin influenced the WHC values of yogurts positively. However, the WHC of yogurts improved as the amount of hazelnut skin grew. A better water-holding property of enriched yogurts may be explained by the high content of the insoluble dietary fibre in the hazelnut skin. This was also confirmed by other authors, who found that the WHC of yogurts was increased by addition of rice bran [34]; yogurts with buckwheat and spelt hulls had lower syneresis values as compared with the control [16].

The colour parameters of enriched yogurts differed with the addition of hazelnut skin due to its pigmentation. The L* values of control yogurts were significantly higher than those of yogurts enriched with hazelnut skin (*p* < 0.05). The L* values decreased gradually along with increases in the amount of hazelnut skin. A similar reduction in L* value was also reported for other kinds of functional yogurts, such as fortified yogurt with rice bran [34], fortified yogurt with pineapple peel powder [12], and fortified yogurt with buckwheat and spelt hull [16]. The addition of hazelnut skin to yogurts increased the red colour ratio compared to the control but did not cause any change in b* values. As the hazelnut skin ratio increases, the decrease in whiteness, and the increase in redness and colour intensity are the expected results. The high proportion of the red colour that increases with the dose of hazelnut skin may be due to the high phenol content in the hazelnut skin. The ΔE value of enriched yogurts showed that the colour changes due to the addition of hazelnut skin are significant. Similarly, Hanafi et al. (2021) [27] found that coconut residue addition to probiotic ice cream increased the ΔE value and affected the colour significantly.

An increase in proteolytic activity was observed, especially after day 7 of storage. The reason for this is thought to be due to the increase of yogurt bacteria and the ongoing acid production during storage. In parallel with our findings, Donkor et al. (2006) [28] found that the increase in proteolytic activity was significant (*p* < 0.05) during storage, except for day 1. The highest levels of proteolytic activity were found in the enriched yogurts on days 7 and 21 and found in the control yogurt at the end of the storage.

TPC also increased with increasing hazelnut skin ratio. However, prolonged storage did not cause any significant differences (*p* > 0.05) in TPC for control yogurts, while fluctuations occurred for enriched yogurts. The fluctuation may result from the decomposition of polymeric phenolics in the presence of the lactic acid bacteria during storage. The highest TPC value was observed in the yogurt enriched with 4% hazelnut skin on day 7 of storage. In addition, the lowest value was observed in control yogurts on days 1 and 7. Bertolino et al. (2015) [6] had similar results, indicating that hazelnut skin enrichment increased the TPC in yogurts. Similar to our results, they reported that TPC increased as the hazelnut skin ratio increased. Iron is necessary for oxygen transport, respiration, and the activity of many enzymes. On the other hand, reduced iron may potentate oxygen toxicity by converting the less reactive hydrogen peroxide to the more reactive oxygen species. Therefore, minimizing the Fe^2+^ concentration protects against oxidative damage [30]. All enriched yogurts showed more chelating activity on ferrous ions than the control yogurts during storage. There are significant differences between yogurt groups (*p* < 0.05). As the amount of hazelnut skin in yogurts increased, the chelating activity also increased. During the 60 min incubation period, yogurts enriched with 4% hazelnut skin had the highest values, approximately double the correspondent values of the control samples. In addition, the increase in incubation time significantly (*p* < 0.05) improved the iron-chelating activities of all yogurt groups except the control. Hazelnut skin has been previously reported to show antioxidant activity due to the presence of condensed tannins [4]. In addition, milk protein proteolysis and organic acid production resulting from microbial metabolic activity during fermentation and cold storage may have contributed to the increase in antioxidant activity [5]. The studies showed that enrichment of yogurts with appropriate by-products or waste food such as rice bran, cactus pear peel, and coriander and cumin seeds may increase antioxidant activity [5,34,36].

Yogurts enriched with hazelnut skin had similar sensory scores compared to control yogurts, without negative effects. Contrary to our study, Demirci et al. (2017) [34] found that all rice-bran-enriched yogurts (1, 2, 3%) had lower sensory scores than control yogurts. Bertolino et al. (2015) [6] reported that the type of hazelnut used and the amount of hazelnut skin affected consumer preferences differently. In addition, the sensory preferences of consumers may differ according to the waste product used for enrichment and the amount used.

## 5. Conclusions

Yogurts with hazelnut skin differed with regard to the physicochemical properties. The enrichment of yogurts with hazelnut skin caused an increase in TS and fat content. In general, the pH values of yogurts enriched with hazelnut skin were higher than control yogurts, and the titratable acidity decreased as the amount of hazelnut skin in yogurts increased. Hazelnut skin added to yogurts had an impact on the survival of yogurt bacteria, and their viability in enriched yogurts was higher than the control. In our experiments, the highest viable counts were enumerated in yogurts enriched with 4% hazelnut skin on day 21. However, the addition of hazelnut skin decreased the textural properties and apparent viscosity of yogurts. The enrichment with hazelnut skin contributed to the WHC as well as the antioxidant capacity of the final product; as the amount of added hazelnut skin increased, its contribution also increased. Overall, lower instrumental texture parameters and apparent viscosity values were observed in enriched yogurts. In addition, enriched yogurts had similar sensorial properties to control yogurts, which is promising in terms of consumer acceptability.

Consequently, this study demonstrated that hazelnut skin, which is a food by-product, can be utilized with good results to produce functional yogurt. Further studies will focus on optimal amounts to be used in yogurt recipes, depending on the quality of the raw material and TRL (technology readiness level) increment for this new product development.

## Figures and Tables

**Table 1:** Physicochemical parameters of yogurts enriched with hazelnut skin.

Sample	Item		
	TS, %	Fat, %	Protein, %
K	16.49 ± 0.07 ^B^	3.45 ± 0.03 ^D^	5.05 ± 0.07 ^A^
A	16.99 ± 0.05 ^AB^	4.03 ± 0.03 ^C^	4.68 ± 0.05 ^A^
B	17.36 ± 0.05 ^AB^	4.31 ± 0.03 ^B^	4.68 ± 0.05 ^A^
C	17.68 ± 0.12 ^A^	4.60 ± 0.03 ^A^	4.63 ± 0.12 ^A^

^A–D^ Means ± standard deviations for the same storage day with different superscript uppercase letters are significantly different (*p* < 0.05). TS = total solid, K = control yogurt without hazelnut skin, A = yogurt enriched with 2% hazelnut skin, B = yogurt enriched with 3% hazelnut skin, C = yogurt enriched with 4% hazelnut skin.

**Table 2:** pH, lactic acid (%), and viable bacteria count (log CFU/g) changes in yogurts enriched with hazelnut skin during storage.

Item	Sample	Storage Days				
		1	7	14	21	28
pH	K	4.41 ± 0.01 ^Ba^	4.21 ± 0.00 ^Bab^	4.17 ± 0.01 ^BCb^	4.05 ± 0.02 ^Cb^	4.21 ± 0.18 ^Aab^
	A	4.56 ± 0.06 ^Aba^	4.21 ± 0.00 ^Bb^	4.07 ± 0.05 ^Cc^	4.09 ± 0.01 ^BCc^	4.08 ± 0.01 ^Ac^
	B	4.57 ± 0.10 ^Aba^	4.35 ± 0.15 ^Abab^	4.29 ± 0.12 ^Abb^	4.13 ± 0.03 ^Bb^	4.07 ± 0.00 ^Ab^
	C	4.64 ± 0.01 ^Aa^	4.59 ± 0.08 ^Aa^	4.39 ± 0.00 ^Ab^	4.39 ± 0.00 ^Ab^	4.18 ± 0.02 ^Ac^
Lactic acid (%)	K	1.39 ± 0.04 ^Ab^	1.38 ± 0.00 ^Ab^	1.47 ± 0.02 ^Aa^	1.54 ± 0.00 ^Aa^	1.48 ± 0.03 ^Aa^
	A	1.22 ± 0.02 ^Bb^	1.36 ± 0.02 ^Aa^	1.35 ± 0.02 ^Aba^	1.24 ± 0.03 ^Bb^	1.27 ± 0.03 ^Bb^
	B	1.15 ± 0.00 ^BCb^	1.22 ± 0.06 ^Bb^	1.46 ± 0.02 ^Aa^	1.17 ± 0.00 ^Cb^	1.18 ± 0.00 ^Cb^
	C	1.09 ± 0.03 ^Cab^	1.04 ± 0.04 ^Cb^	1.22 ± 0.10 ^Ba^	0.98 ± 0.01 ^Db^	0.99 ± 0.02 ^Db^
*Lactobacillus delbrueckii* subsp. *bulgaricus*	K	9.05 ± 0.04 ^Ba^	8.93 ± 0.02 ^Aa^	8.64 ± 0.12 ^Bb^	9.06 ± 0.03 ^Ca^	8.67 ± 0.04 ^ABb^
	A	8.61 ± 0.01 ^Cd^	8.67 ± 0.04 ^Bcd^	8.84 ± 0.09 ^ABb^	9.24 ± 0.00 ^Ba^	8.77 ± 0.01 ^Abc^
	B	9.09 ± 0.03 ^ABa^	8.92 ± 0.00 ^Ab^	8.97 ± 0.00 ^Ab^	9.10 ± 0.01 ^Ca^	8.72 ± 0.04 ^Ac^
	C	9.18 ± 0.02 ^Aa^	8.75 ± 0.02 ^Bb^	8.87 ± 0.07 ^ABb^	9.32 ± 0.01 ^Aa^	8.52 ± 0.10 ^Bc^
*Streptococcus thermophilus*	K	8.61 ± 0.04 ^Bc^	8.76 ± 0.00 ^Bb^	8.74 ± 0.05 ^Bb^	8.89 ± 0.00 ^Ba^	8.52 ± 0.03 ^Ac^
	A	8.37 ± 0.08 ^BCc^	8.68 ± 0.00 ^Cb^	8.57 ± 0.04 ^Cb^	8.87 ± 0.00 ^Ca^	8.62 ± 0.03 ^Ab^
	B	8.30 ± 0.18 ^Cc^	8.76 ± 0.01 ^Bab^	8.86 ± 0.00 ^Aa^	8.79 ± 0.00 ^Dab^	8.58 ± 0.00 ^Ab^
	C	8.97 ± 0.00 ^Aa^	8.82 ± 0.02 ^Ab^	8.79 ± 0.03 ^Abb^	8.97 ± 0.00 ^Aa^	8.36 ± 0.02 ^Bc^

^a–d^ Means ± standard deviations in the same row with different superscript lowercase letters are significantly different (*p* < 0.05); ^A–D^ Means ± standard deviations in the same column with different superscript uppercase letters are significantly different (*p* < 0.05); K = control yogurt without hazelnut skin, A = yogurt enriched with 2% hazelnut skin, B = yogurt enriched with 3% hazelnut skin, C = yogurt enriched with 4% hazelnut skin.

**Table 3:** Hardness, adhesive force, consistency, viscosity index, and apparent viscosity values of yogurts enriched with hazelnut skin during storage.

Item	Sample	Storage Days				
		1	7	14	21	28
Hardness	K	299.63 ± 15.02 ^Ab^	333.88 ± 3.71 ^Aa^	337.88 ± 19.26 ^Aa^	341.63 ± 3.00 ^Aa^	346.00 ± 0.70 ^Aa^
	A	264.63 ± 6.89 ^Aab^	296.00 ± 19.44 ^Bab^	264.75 ± 20.50 ^Bb^	283.50 ± 7.42 ^Ba^	273.13 ± 12.90 ^Bab^
	B	271.63 ± 18.91 ^Aa^	240.00 ± 24.04 ^Ba^	262.00 ± 5.30 ^Ba^	263.50 ± 15.20 ^BCa^	271.13 ± 0.53 ^Ba^
	C	222.50 ± 16.61 ^Ba^	243.50 ± 4.94 ^Ba^	254.75 ± 27.57 ^Ba^	224.38 ± 24.21 ^Ca^	220.75 ± 47.37 ^Ba^
Adhesive force	K	68.37 ± 3.00 ^Aa^	75.37 ± 7.60 ^Aa^	71.12 ± 8.30 ^Aa^	78.37 ± 1.94 ^Aa^	74.37 ± 2.29 ^Aa^
	A	62.62 ± 2.65 ^ABa^	64.62 ± 2.65 ^ABa^	65.00 ± 9.54 ^Aa^	70.00 ± 1.06 ^ABa^	68.25 ± 1.76 ^ABa^
	B	66.00 ± 6.01 ^Aa^	55.25 ± 5.65 ^Ba^	57.75 ± 0.35 ^Aa^	59.25 ± 3.88 ^Ba^	62.50 ± 2.12 ^ABa^
	C	51.50 ± 4.94 ^Ba^	55.12 ± 4.06 ^Ba^	62.37 ± 7.24 ^Aa^	47.12 ± 6.89 ^Ca^	51.25 ± 11.66 ^Ba^
Consistency	K	32.63 ± 1.46 ^Aa^	36.26 ± 0.53 ^Aa^	35.04 ± 5.47 ^Aa^	38.05 ± 0.30 ^Aa^	37.66 ± 0.56 ^Aa^
	A	27.92 ± 0.72 ^Bb^	29.17 ± 1.19 ^Bab^	28.79 ± 2.20 ^Aab^	31.58 ± 0.51 ^Ba^	31.17 ± 0.20 ^ABa^
	B	28.92 ± 0.54 ^Ba^	24.60 ± 4.30 ^Ba^	28.91 ± 0.38 ^Aa^	29.45 ± 3.31 ^BCa^	31.19 ± 0.54 ^ABa^
	C	24.32 ± 1.72 ^Ca^	26.23 ± 1.03 ^Ba^	27.88 ± 2.11 ^Aa^	24.83 ± 1.93 ^Ca^	24.12 ± 5.86 ^Ba^
Viscosity Index	K	7.45 ± 0.23 ^Aa^	7.17 ± 0.91 ^Aa^	6.67 ± 1.41 ^Aa^	7.01 ± 0.30 ^ABa^	6.84 ± 0.07 ^Aa^
	A	6.60 ± 0.02 ^ABa^	7.31 ± 0.28 ^Aa^	6.98 ± 0.98 ^Aa^	7.59 ± 0.04 ^Aa^	7.49 ± 0.03 ^Aa^
	B	7.18 ± 0.53 ^Aab^	6.14 ± 0.24 ^Ab^	6.33 ± 0.12 ^Aab^	6.99 ± 0.64 ^ABab^	7.24 ± 0.04 ^Aa^
	C	5.42 ± 0.98 ^Ba^	6.31 ± 0.73 ^Aa^	6.60 ± 1.04 ^Aa^	5.57 ± 0.93 ^Ba^	5.88 ± 1.50 ^Aa^
Apparent viscosity	K	4,86 ± 0.00 ^Ac^	5.22 ± 0.13 ^Abc^	4.46 ± 0.00 ^Ab^	5.17 ± 0.02 ^Abc^	5.94 ± 0.34 ^Aa^
	A	4,02 ± 0.42 ^ABa^	4.91 ± 0.26 ^Aa^	5.25 ± 0.19 ^Aa^	5.18 ± 0.02 ^Aa^	5.34 ± 0.11 ^ABa^
	B	4,21 ± 0.34 ^ABb^	5.16 ± 0.17 ^Aa^	4.48 ± 0.31 ^Bb^	4.73 ± 0,18 ^Aab^	4.75 ± 0.00 ^Bab^
	C	3,47 ± 0.24 ^Ba^	3.71 ± 0.11 ^Ba^	3.28 ± 0.11 ^Ca^	3.20 ± 0.35 ^Aa^	3.81 ± 0.37 ^Ca^

^a–c^ Means ± standard deviations in the same row with different superscript lowercase letters are significantly different (*p* < 0.05); ^A–C^ Means ± standard deviations in the same column with different superscript uppercase letters are significantly different (*p* < 0.05).

**Table 4:** Colour-space parameters of yogurts enriched with hazelnut skin during storage.

Item	Sample	Storage Days				
		1	7	14	21	28
L*	K	87.06 ± 0.76 ^Aa^	89.81 ± 0.08 ^Aa^	89.28 ± 0.28 ^Aa^	88.31 ± 0.78 ^Aa^	88.97 ± 2.75 ^Aa^
	A	68.43 ± 1.95 ^Ba^	72.94 ± 0.87 ^Ba^	69.38 ± 1.27 ^Ba^	68.88 ± 3.73 ^Ba^	73.04 ± 1.46 ^Ba^
	B	63.44 ± 0.42 ^Cb^	66.96 ± 0.27 ^Ca^	58.06 ± 1.11 ^Cc^	59.75 ± 1.34 ^Cc^	63.68 ± 1.52 ^Cb^
	C	57.87 ± 1.81 ^Dbc^	62.64 ± 3.07 ^Ca^	59.15 ± 0.93 ^Cabc^	55.01 ± 0.03 ^Cc^	62.20 ± 1.44 ^Cab^
A*	K	−3.34 ± 0.01 ^Da^	−3.43 ± 0.02 ^Da^	−3.38 ± 0.14 ^Ca^	−3.54 ± 0.06 ^Da^	−3.39 ± 0.16 ^Da^
	A	5.72 ± 0.13 ^Ca^	5.52 ± 0.11 ^Cab^	5.69 ± 0.06 ^Ba^	5.57 ± 0.03 ^Cab^	5.36 ± 0.15 ^Cb^
	B	7.28 ± 0.02 ^Ba^	7.37 ± 0.01 ^Ba^	7.35 ± 0.56 ^Aa^	7.03 ± 0.34 ^Ba^	7.12 ± 0.04 ^Ba^
	C	8.01 ± 0.08 ^Aa^	8.08 ± 0.34 ^Aa^	8.02 ± 0.24 ^Aa^	7.73 ± 0.04 ^Aa^	7.77 ± 0.08 ^Aa^
B*	K	9.23 ± 0.17 ^Aab^	9.37 ± 0.17 ^Aa^	9.45 ± 0.20 ^Aa^	9.29 ± 0.16 ^Aab^	8.85 ± 0.15 ^Ab^
	A	9.69 ± 0.48 ^Aa^	10.41 ± 0.81 ^Aa^	10.03 ± 0.70 ^Aa^	9.63 ± 1.38 ^Aa^	9.94 ± 0.42 ^Aa^
	B	9.98 ± 0.10 ^Ab^	10.94 ± 0.25 ^Aa^	8.09 ± 0.43 ^Bc^	8.42 ± 0.15 ^Ac^	8.85 ± 0.53 ^Ac^
	C	9.42 ± 0.75 ^Aab^	10.56 ± 1.30 ^Aa^	9.75 ± 0.03 ^Aa^	7.80 ± 0.16 ^Ab^	9.75 ± 0.48 ^Aa^
Chroma (C*)	K	10.60 ± 0.96 ^Aa^	8.69 ± 0.16 ^Ba^	9.40 ± 0.65 ^Ca^	9.94 ± 0.17 ^Aa^	9.47 ± 0.19 ^Ca^
	A	10.26 ± 0.43 ^Aa^	11.78 ± 0.77 ^Aa^	11.52 ± 0.64 ^ABa^	11.13 ± 1.18 ^Aa^	11.29 ± 0.29 ^Ba^
	B	12.35 ± 0.09 ^Aa^	13.17 ± 0.22 ^Aa^	10.92 ± 0.70 ^BCb^	10.96 ± 0.09 ^Ab^	11.35 ± 0.39 ^Bb^
	C	12.36 ± 0.63 ^Aab^	13.29 ± 1.24 ^Aa^	12.62 ± 0.12 ^Aab^	10.97 ± 0.08 ^Ab^	12.47 ± 0.32 ^Aab^
∆E	K					
	A	20.72 ± 0.41 ^Ca^	19.13 ± 0.35 ^Ca^	21.88 ± 0,37 ^Ba^	21.46 ± 0.43 ^Ca^	18.21 ± 0.34 ^Ca^
	B	25.91 ± 0.51 ^Bc^	25.32 ± 0.46 ^Bc^	33.04 ± 0,60 ^Aa^	30.47 ± 0.62 ^Bab^	27.39 ± 0.56 ^Bb^
	C	31.32 ± 0.59 ^Ab^	29.53 ± 0.53 ^Ab^	32.22 ± 0,64 ^Aab^	35.19 ± 0.69 ^Aa^	29.02 ± 0.53 ^Ab^

^a–c^ Means ± standard deviations in the same row with different superscript lowercase letters are significantly different (*p* < 0.05); ^A–D^ Means ± standard deviations in the same column with different superscript uppercase letters are significantly different (*p* < 0.05).

## Data Availability

The datasets generated for this study are available on request to the corresponding author.
